# Dissipative particle dynamics model of homogalacturonan based on molecular dynamics simulations

**DOI:** 10.1038/s41598-020-71820-2

**Published:** 2020-09-07

**Authors:** P. M. Pieczywek, W. Płaziński, A. Zdunek

**Affiliations:** 1 Institute of Agrophysics, Polish Academy of Sciences, Doświadczalna 4, 20-270 Lublin, Poland; 2 Jerzy Haber Institute of Catalysis and Surface Chemistry, Polish Academy of Sciences, Niezapominajek 8, 30-239 Cracow, Poland

**Keywords:** Fluid dynamics, Polysaccharides

## Abstract

In this study we present an alternative dissipative particle dynamics (DPD) parametrization strategy based on data extracted from the united-atom molecular simulations. The model of the homogalacturonan was designed to test the ability of the formation of large-scale structures via hydrogen bonding in water. The extraction of coarse-grained parameters from atomistic molecular dynamics was achieved by means of the proposed molecule aggregation algorithm based on an iterative nearest neighbour search. A novel approach to a time-scale calibration scheme based on matching the average velocities of coarse-grained particles enabled the DPD forcefield to reproduce essential structural features of homogalacturonan molecular chains. The successful application of the proposed parametrization method allowed for the reproduction of the shapes of radial distribution functions, particle velocities and diffusivity of the atomistic molecular dynamics model using DPD force field. The structure of polygalacturonic acid molecules was mapped into the DPD force field by means of the distance and angular bond characteristics, which closely matched the MD results. The resulting DPD trajectories showed that randomly dispersed homogalacturonan chains had a tendency to aggregate into highly organized 3D structures. The final structure resembled a three-dimensional network created by tightly associated homogalacturonan chains organized into thick fibres.

## Introduction

Dissipative particle dynamics (DPD) is a stochastic mesoscale particle model, commonly used for the large-scale modelling of self-assembly of the polymeric systems^1^. First introduced by Hoogerbrugge and Koelman^2^ DPD was further refined by Español and Warren^3^. Compared to methods based on atomistic force fields such as molecular dynamics (MD), DPD permits the simulation of large systems over relatively long-time scales^4^. This is achieved by the utilization of simplified soft potentials and coarse-grained representations of modelled structures. DPD models have proven to be useful in many fields of chemical research, enabling the description of complex phenomena of self-assembly of soft-matter quasicrystals and their approximants^5^, spontaneous fibril formation by random-coil peptides^6^, and simulations of large-scale micellar structures^7,8^. In contrast to MD, the physical attributes of the soft matter such as actual atomic diameter, mass, energy, and depth of the potential well cannot be set in the DPD formulation^9^.

In DPD systems the intended physical properties are determined by means of parameter calibration. The most popular method of calibration was introduced by Groot and Warren^10^ through mapping onto Flory–Huggins theory. Other researchers coupled DPD with MD simulations to calibrate models by matching the structural data from the atomistic simulations^11,12^. Keaveny et al.^13^ showed an MD-based calibration scheme designed for the parametrization of models suitable for flow problems. However, most of the presented methods were intended to aim at a specific system property, which lead to situations where other statistics of the simulated system were lost. More recently Li et al.^14^ presented a bottom-up coarse-graining procedure to construct mesoscopic force fields directly from microscopic dynamics. The dissipative particle dynamics model was derived from the Mori–Zwanzig (MZ) projection of the underlying atomistic dynamics. The DPD models introduced included the radial and transverse force components between the beads, and the rotational motion of the particles. Quantitative comparisons between these DPD models indicated that the DPD models with MZ-guided force fields were able to reproduce the velocity autocorrelation function as well as the pair correlation function of the MD system.

In this study we present an alternative parametrization strategy of standard DPD force field based on data extracted from a united-atom molecular dynamics model and solving an inverse problem targeting certain properties. The presented methodology leads to systems that reproduce the shapes of radial distribution functions, particle velocities and the diffusivity of the CG water model and also adequately simulate the structures of reference models of polymers. The presented approach is based on a case study of basic pectic polysaccharide – homogalacturonan. Homogalacturonan (HG) is the most abundant form of plant cell wall pectic polysaccharide. The unsubstituted backbone of HG consists of d-galacturonate (GalA) units joined in chains by α-(1 → 4) glycosidic linkages. It is known for its gelling properties which have been partially attributed to an ability to form Ca^2+^ cross-links^15,16^. Some studies have also suggested that homogalacturonan can aggregate due to weak hydrogen bonding via carboxyl groups^17,18^. Until now, molecular studies of pectic polysaccharides have been limited to the interaction of two or three molecular chains with up to 20 residues^19–23^ or conformations of single chains consisting of 50 residues^24^.

Currently no cell wall pectic polysaccharide models are available that would allow for the simulation of their aggregation and large-scale clusters. Therefore, the aim of this study was to provide a fully parametrized coarse-grained model of homogalacturonan based on a dissipative particle dynamics force field. Parametrization was based on atomistic molecular dynamics models, which are coarse-grained and make use of a newly introduced algorithm. The model was designed to test the ability of the formation of large-scale structures via the hydrogen bonding of homogalacturonan in water solutions.

## Materials and methods

### Model system

**Figure 1:**
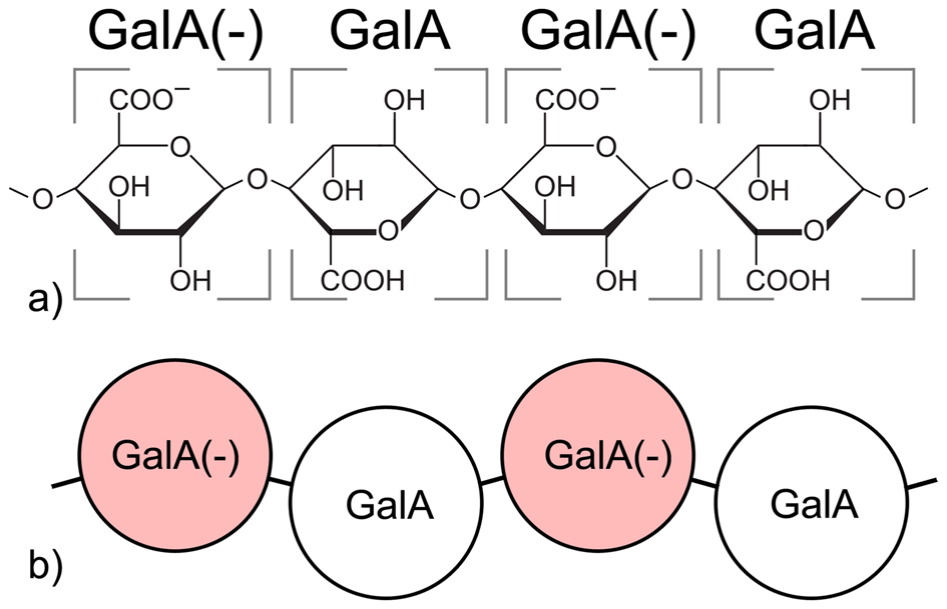
Molecular structure of homogalacturonan with the two possible protonation levels of the carboxyl groups of galacturonic acid units (**a**) and the corresponding coarse-grained representation of homogalacturonan molecular chains (**b**).

The unsubstituted backbone of homogalacturonan (HG) consisting of galacturonic acid units joined in chains by α-(1 → 4) glycosidic linkages was chosen as the model system. In order to test the hypothesis of HG aggregation by hydrogen bonding, the galacturonic acid units were simulated in two states of the protonation of carboxyl groups—protonated/undissociated (GalA) and deprotonated/dissociated (GalA(−)). The homogalacturonan chains were simulated with all of the carboxyl groups being protonated or deprotonated, as well as half of the GlaA units being protonated and half deprotonated (Fig. 1). Figure 1 shows the level of generalization of the HG backbone adopted in this study.

### DPD simulation

An extensive overview of the dissipative particle dynamics simulation method has already been provided by other researchers^25,26^ therefore, in this section only the essential mathematical formulations of DPD are presented. In brief, the DPD coarse-grained particles, called beads, represent clusters of atoms which have a state that evolves in time according to Newton’s equations of motion. Each bead *i* interacts with the neighbouring bead *j* via three basic non-bonded interaction forces, a conservative force ${\overrightarrow{{\varvec{F}}}}_{ij}^{C}$, a dissipative force ${\overrightarrow{\mathrm{F}}}_{\mathrm{ij}}^{\mathrm{D}}$ and a random force ${\overrightarrow{\mathrm{F}}}_{\mathrm{ij}}^{\mathrm{R}}$. These forces can act within a specific length range , called the cut-off radius. The interaction forces are given by:

1 $$\overrightarrow{{\mathrm{F}}_{\mathrm{ij}}^{\mathrm{C}}}={\mathrm{a}}_{\mathrm{ij}}{\upomega }^{\mathrm{C}}\left({\mathrm{r}}_{\mathrm{ij}}\right){\widehat{\mathrm{r}}}_{\mathrm{ij}}$$

2 $${\overrightarrow{\mathrm{F}}}_{\mathrm{ij}}^{\mathrm{D}}=-\upgamma {\upomega }^{\mathrm{D}}\left({\mathrm{r}}_{\mathrm{ij}}\right)\left({\overrightarrow{\mathrm{v}}}_{\mathrm{ij}}\bullet {\widehat{\mathrm{r}}}_{\mathrm{ij}}\right){\widehat{\mathrm{r}}}_{\mathrm{ij}}$$

3 $${\overrightarrow{\mathrm{F}}}_{\mathrm{ij}}^{\mathrm{R}}=\upsigma {\upomega }^{\mathrm{R}}\left({\mathrm{r}}_{\mathrm{ij}}\right){\upxi }_{\mathrm{ij}}{\Delta \mathrm{t}}^{-1/2}{\widehat{\mathrm{r}}}_{\mathrm{ij}}$$

where ${\overrightarrow{\mathrm{r}}}_{\mathrm{ij}}={\overrightarrow{\mathrm{r}}}_{\mathrm{i}}-{\overrightarrow{\mathrm{r}}}_{\mathrm{j}}$, ${\mathrm{r}}_{\mathrm{ij}}=\left|{\overrightarrow{\mathrm{r}}}_{\mathrm{ij}}\right|$, ${\widehat{\mathrm{r}}}_{\mathrm{ij}}={\overrightarrow{\mathrm{r}}}_{\mathrm{ij}}/{\mathrm{r}}_{\mathrm{ij}}$, and ${\overrightarrow{\mathrm{r}}}_{\mathrm{i}}$, ${\overrightarrow{\mathrm{r}}}_{\mathrm{j}}$ are the position vectors of bead *i* and *j*, respectively. ${\overrightarrow{\mathrm{v}}}_{\mathrm{ij}}={\overrightarrow{\mathrm{v}}}_{\mathrm{i}}-{\overrightarrow{\mathrm{v}}}_{\mathrm{j}}$ is the relative velocity between two beads, calculated from their velocity vectors ${\overrightarrow{\mathrm{v}}}_{\mathrm{i}}$ and ${\overrightarrow{\mathrm{v}}}_{\mathrm{j}}$. The ${\mathrm{a}}_{\mathrm{ij}}$ symbol from the conservative force equation stands for the repulsion constant between two interacting beads. $\gamma$ and $\upsigma$ are the corresponding symbols for the dissipative and random forces constant coefficients, respectively. ${\upxi }_{\mathrm{ij}}$ is a value from a set of random numbers with zero mean and unit variance, independently chosen for each interacting pair *ij.* Each type of interaction force is scaled by its own weight function. ${\upomega }^{\mathrm{C}}$, ${\upomega }^{\mathrm{D}}$ and ${\upomega }^{\mathrm{R}}$ are the weight functions for conservative, dissipative and random forces, respectively. The weight function for conservative force is defined by the following simple linear relationship:

4 $$ {\upomega }^{{\text{C}}} \left( {{\text{r}}_{{{\text{ij}}}} } \right) = \left\{ {\begin{array}{*{20}l} {\left( {1 - {\text{r}}_{{{\text{ij}}}} /{\text{r}}_{{\text{C}}} } \right)} \hfill & {{\text{when}}\,{\text{r}}_{{{\text{ij}}}} < {\text{r}}_{{\text{C}}} } \hfill \\ 0 \hfill & {{\text{when }}\,{\text{r}}_{{{\text{ij}}}} > {\text{r}}_{{\text{C}}} } \hfill \\ \end{array} } \right.. $$

The generalized form of the weight function for dissipative forces is defined as:

5 $$ {\upomega }^{{\text{D}}} \left( {{\text{r}}_{{{\text{ij}}}} } \right) = \left\{ {\begin{array}{*{20}l} {\left( {1 - {\text{r}}_{{{\text{ij}}}} /{\text{r}}_{{\text{C}}} } \right)^{{\text{S}}} } \hfill & {{\text{when}}\, {\text{r}}_{{{\text{ij}}}} < {\text{r}}_{{\text{C}}} } \hfill \\ 0 \hfill & {{\text{when}}\,{\text{r}}_{{{\text{ij}}}} > {\text{r}}_{{\text{C}}} } \hfill \\ \end{array} } \right. $$

where the exponent *s* modifies the shape of the weight function and is modified to adjust the fluid viscosity^27,28^. In order to obey the Boltzmann statistics and achieve a well-defined equilibrium state in temperature *T*, the following equations must be fulfilled:

6 $${\upomega }^{\mathrm{D}}\left({\mathrm{r}}_{\mathrm{ij}}\right)={\left[{\upomega }^{\mathrm{R}}\left({\mathrm{r}}_{\mathrm{ij}}\right)\right]}^{2}$$

7 $${\upsigma }^{2}=2\upgamma {\mathrm{k}}_{\mathrm{B}}\mathrm{T}$$

where ${\mathrm{k}}_{\mathrm{B}}$ is the Boltzmann constant. In order to simulate polysaccharide molecular chains, the adjacent beads must be constrained with permanent lengths and angular bonds. In this study, the bonds were modelled using harmonic spring quadratic potentials given as:

8 $${\mathrm{u}}_{\mathrm{ij}}^{\mathrm{B}}={\frac{1}{2}\mathrm{k}}_{\mathrm{S}}{\left({\mathrm{r}}_{\mathrm{ij}}-{\mathrm{l}}_{0}\right)}^{2}$$

9 $${\mathrm{u}}_{\mathrm{ij}}^{\mathrm{B}}={\frac{1}{2}\mathrm{k}}_{\mathrm{A}}{\left({\uptheta }_{\mathrm{ijk}}-{\uptheta }_{0}\right)}^{2}$$

where ${\mathrm{l}}_{0}$ and ${\theta }_{0}$ are the equilibrium lengths and angles for particles *i*, *j* and *k*. The stiffness of the length and angular bond constraints is defined by the values of ${\mathrm{k}}_{\mathrm{S}}$ and ${\mathrm{k}}_{\mathrm{A}}$.

### Molecular dynamics simulation details

All MD simulations were carried out with the GROMACS 5.0 package^29^. The force field parameters used for the simulations were adopted from the GROMOS 56A6_CARBO_ force field^30,31^ whereas the interactions involving carboxylate moieties were described in terms of the recently proposed extension of GROMOS 56A6_CARBO_^22^. The molecular systems under consideration were placed in cubic simulation boxes filled with the appropriate number of simple point charge (SPC) water molecules^32^. The unbiased simulations were carried out under periodic boundary conditions and with an isothermal-isobaric ensemble. The temperature was maintained close to its reference value (298 K) by applying the V-rescale thermostat^33^, whereas for constant pressure (1 bar, isotropic coordinate scaling) the Parrinello-Rahman barostat^34^ was used with a relaxation time of 0.4 ps. The equations of motion were integrated with a time step of 2 fs using the leap-frog scheme35. The solute bond lengths were constrained by the application of the LINCS procedure with a relative geometric tolerance of 10^–4^^36,37^. The full rigidity of the water molecules was enforced by the application of the SETTLE procedure ^32^. The translational centre-of-mass motion was removed for every time step separately for the solute and the solvent. The non-bonded interactions were calculated using a twin-range scheme 38, with short- and long-range cut-off distances set to 0.8 and 1.4 nm, respectively, and an update frequency of 5 time steps for the short-range pair list and intermediate-range interactions. The reaction-field correction was applied to account for the mean effect of the electrostatic interactions beyond the long-range cut off distance, using a relative dielectric permittivity of 61 as appropriate for the SPC water model ^39–41^. All of the systems were preoptimized by a 0.5–1.5 ns constant-pressure MD equilibration at 1 bar and 298 K, thereby ensuring an effective solvent density appropriate for these conditions in the subsequent production simulations. After equilibration, all unbiased simulations were carried out for up to 100 ns and the trajectory was saved every 2 ps for further analysis.

In order to map the characteristics of the atomistic models into the DPD system, a series of molecular dynamics simulations were conducted starting from the basic structural units of homogalacturonan up to full-length molecular chains. Molecular dynamics models were used to carry out the coarse-graining procedure and extract molecular characteristics such as the radial distribution function, velocity of particles and diffusion constant, as well as the distributions of lengths and angles for molecules bonded with length and angular bonds. The initial DPD calibration was carried out based on a simple model of water consisted of 967 SPC water molecules in a 31.036 × 31.036 × 31.036 Å cubic simulation box. The interactions of galacturonic acid with the surrounding water molecules were tackled based on the simulation of a single galacturonic acid molecule model in a water box. Two variations of such a model were used, one with a dissociated and one with an undissociated carboxyl functional group. The non-bonded interactions between the galacturonic acid molecules were extracted from three models: one with 120 deprotonated galacturonic acid molecules placed in a water box, one with 120 protonated galacturonic acid molecules placed in a water box, and the last one with 120 galacturonic acid molecules in water box with half of the molecules being protonated and half deprotonated. The interactions between the bonded molecules were parameterized based on simulations which involved homogalacturonan molecular chains with different states of carboxyl groups. Two simulations of the molecular chains consisting of eight galacturonic acid units were performed, one with all of the carboxyl groups being dissociated and one with all of the carboxyl groups undissociated. Next, two homogalacturonan chains with 26 units of galacturonic acid were simulated. In both models, half of carboxyl groups were deprotonated and half of them were protonated, but they were arranged in two different patterns. In the first model, the dissociated and undissociated carboxyl groups alternated one after the other (Fig. 1b). In the second model, the dissociated and undissociated carboxyl groups were arranged in alternating pairs, two dissociated units next to two undissociated ones. The homogalacturonan chain simulations allowed for the determination of the characteristics of the bond lengths for three possible combinations of neighbouring galacturonic acid molecules, namely [GalA][GalA], [GalA(−)][GalA(−)] and [GalA(−)][GalA]. The angular bonds were parametrized for six possible combinations: [GalA][GalA][GalA], [GalA(−)][GalA(−)][GalA], [GalA(−)][GalA][GalA], [GalA(−)][GalA(−)][GalA(−)], [GalA][GalA(−)][GalA], [GalA(−)][GalA][GalA(−)].

### Extraction of coarse-grained parameters from MD simulations

As in other CG methods, beads from the DPD models correspond to coarse-grained molecules that numerically represent clusters of individual atoms. CG methods require a systematic coarse-graining approach, which leads to the reproduction of the desired properties of the atomistic model in a less detailed system, thus enabling more efficient calculations. For the CG representations of bonded atoms several mapping schemes have been used to date. Most commonly, multiple heavy atoms are grouped based on their specific functional groups and then characterized by the force field associated with their centre-of-mass^42–44^.

However, in the case of atomistic water models, where the water particles move independently, groups/clouds of CG molecules have to be dynamically identified. Therefore, a clustering method is required to enable the mapping of multiple water molecules into a single CG bead. Recently, Hadley and McCabe^45^ demonstrated a successful approach to mapping multiple water molecules to a single CG bead using the k-means clustering algorithm.

K-means is an unsupervised algorithm that solves the clustering problems. The algorithm requires an a priori knowledge about the number of clusters (k-value) within the data set and their initial “locations” as defined by the mean values of the processed variables. K-means divides observations into k clusters in which each observation belongs to the cluster with the nearest mean/location. Hadley and McCabe applied the k-means algorithm to cluster particles based on their coordinates in space. In their approach, the k-value corresponded to the total number of CG beads in simulated system. In the presented algorithm for each simulation time frame, the water molecules were divided into a predefined number of groups based on their proximity. Next, the positions of clusters from a previous simulation time frame were updated based on the positions of the nearest clusters of water molecules from the currently processed data frame. Although successful, the authors reported difficulties when defining the number of clusters for k-means. They pointed out some issues regarding the stability of the algorithm and inability to converge with the desired number of equally sized clusters. Therefore, they introduced intermediate procedures of clustering with a variable value of k to improve the convergence of the algorithm.

**Figure 2:**
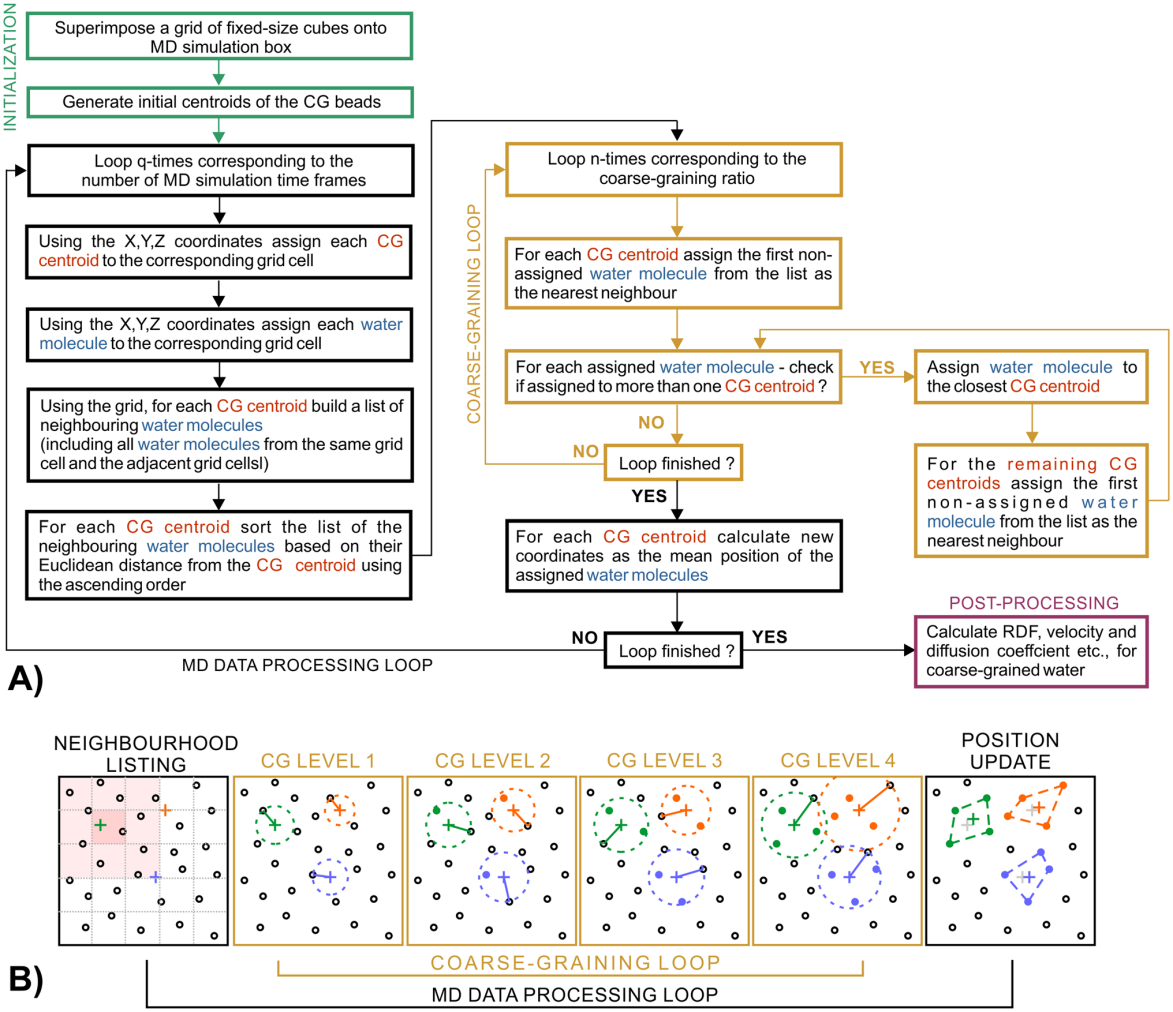
The flow chart of the step-wise iterative clustering algorithm (**A**), with a detailed view of the single data frame processing loop (**B**). The first frame depicts the process of finding adjacent molecules for a single bead marked with a green cross-point. The nearest molecule is searched for in the area adjacent to the unit cell in which the coarse-grained bead is located (marked in red). The next four frames depict the aggregation of water molecules (black circles) into molecular clouds according to their distance from the centre of mass of three coarse-grained beads (coloured cross points). The final frame shows the updated positions of the coarse-grained beads, calculated as the centre of mass of the molecular clouds.

In this study we proposed an alternative step-wise iterative nearest neighbour search algorithm, which solved this issue by forcing the constant and equal number of molecules in all clusters. The flow chart of the clustering procedure is presented in Fig. 2. The major difference between the proposed algorithm and the k-means approach was that instead of the total number of beads in the system, the algorithm was required to provide the number of the water molecules per bead—the CG ratio. The degree of coarse-graining corresponded to the number of clustering steps that the algorithm carried out in each time-step (for instance, for 4 to 1 it results in four steps). The initial positions of the CG beads were defined by randomly chosen coordinates of oxygen atoms from the first frame of the trajectory of the water molecules. Then, for each step of the algorithm, an iterative search for the unique nearest water molecule was carried out for all CG beads. The unique nearest water molecule was defined by means of the Euclidean distance from the centre of mass of a CG bead. Each molecule could be identified as the nearest neighbour to only one centre of mass of a CG bead. If the water molecule was identified as the nearest neighbour for more than one CG bead, then it was assigned to the closest one and the search was carried out again for the remaining beads using a set of non-assigned water molecules during the next iteration (Fig. 2A, coarse-graining loop). Each step of the algorithm finished when all of the beads had one assigned water molecule. The algorithm finished when all of the CG beads had the same number of molecules assigned to them, equal to the degree of coarse-graining. The positions of the beads were updated when all of the sub-steps of the algorithm were finished. The number of beads as well as the number of molecules per bead were fixed. The step-wise procedure reduced the probability of the occurrence of events where the algorithm was unable to find any non-assigned molecule. For the sake of the stability of the algorithm, the desired number of the CG beads was selected in such a way that there were always a few free molecules left in the base system. The aggregation algorithm was used to map three atomistic characteristics into the CG level: the radial distribution function (RDF), the velocity distribution and the diffusivity coefficient of the water beads. The diffusivity of the simulated system was calculated using the standard equation for the diffusion coefficient in molecular dynamics^47^. The same aggregation process of water molecules was carried out to produce simulations of galacturonic acid molecules and homogalacturonan chains in water solutions.

### DPD base units and simulation parameters

In order to extract the CG parameters from MD simulations, firstly the coarse-graining ratio had to be established. In the conventional DPD approach, the diameters and volumes of all of the beads are the same. Therefore, the proposed algorithm of coarse-graining (see section 2.4) had to provide groups of atoms of a similar size (in terms of the number of molecules). In DPD systems the coarse-graining ratio is expressed as the number of water molecules represented by a single DPD bead. In order to reflect the behaviour of homogalacturonan chains in water, a single DPD bead was set to represent one molecule of galacturonic acid (Fig. 1). The volume of the galacturonic acid molecule was estimated to be equal to 140.3 Å^3^ (estimated using Vega ZZ Software^47^). Since the volume occupied by four water molecules (120 Å^3^^25^) was reasonably close to the volume of the galacturonic acid molecule, a four to one coarse-graining ratio was chosen for this system. For galacturonic acid, the position of the corresponding DPD bead was calculated as the centre of mass of this molecule.

To avoid using excessively large or small numbers and to simplify the calculations, the DPD systems were usually scaled by arbitrarily chosen base units. The mass of one water bead consisting of four water molecules ((H_2_O)_4_) equal to 11.968 · 10^–26^ kg, was used as the base mass unit for reduced units while performing DPD simulations. The mass of the corresponding GalA and GalA(−) molecules expressed in DPD reduced units were equal to 2.623 and 2.609, respectively. The MD simulations were performed at 298 K, giving ${\mathrm{k}}_{\mathrm{B}}\mathrm{T}=4.115 \cdot  {10}^{-21}$ J/mol, which was used as the base unit for energy. In most DPD model approaches the cut-off radius of the beads was also used as a base unit for the length scale of the system. In such cases the cut-off radius may be associated with other parameters of the DPD system such as the number of beads *N*, simulation box volume *V* and simulation density number $\uprho$, by a straightforward relationship: ${\mathrm{r}}_{\mathrm{C}}=\sqrt[3]{\frac{\mathrm{\rho V}}{\mathrm{N}}}$. Assuming that the DPD system had to reflect the corresponding MD model, the parameters from the reference water model (section 2.3) were used to deduce the value of ${\mathrm{r}}_{\mathrm{C}}$. The base MD water model consisted of 967 water molecules, therefore the number of corresponding CG molecules was ≈240 (the number was rounded down to leave four free water molecules for the sake of the stability of the CG algorithm described in the previous section). The density number defines the average number of beads per unit cell volume (${\mathrm{r}}_{\mathrm{C}}^{3}$) of the simulation box and significantly affects the output parameters of the system such as viscosity^9^. Usually it is chosen from the range 3–5^48^. In this study, a simulation density number of 3 was chosen, which is most commonly used in DPD simulations^26^. Finally, by putting the volume of the simulation box for the MD water model (29,894 Å^3^) together with the assumed simulation density number and the number of corresponding water beads into the expression for the cut-off radius, the calculated ${\mathrm{r}}_{\mathrm{C}}$ value was found to be equal to 7.2 Å, which was used as the base unit for length.

The limit for the time step size in standard DPD methods is directly connected with the time integration of stochastic dynamics^48^. However, Hafskjold et al.^49^ also showed that even the commonly used time steps can lead to systematic errors in the computed properties within the scope of standard DPD methods. According to their study, errors occurred largely due to the inaccurate integration of the conservative force, namely the numerical truncation of conservative force potential. They concluded that the safest way to avoid these errors is to use small time steps, preferably similar to those used in molecular dynamics. Since this study was aimed at establishing the close correspondence between MD and DPD systems with respect to the basic physical properties, a time step of $\Delta \mathrm{t}=0.001\uptau$ was used to integrate the DPD equations of motion (where $\uptau$ is the base unit of time, which is set experimentally). The exponent *s* from Eq. (5) defines the form of the weight function for dissipative and random forces. It is an intrinsic system parameter that influences the viscosity and diffusion coefficient of the DPD model^50,51^. The ratio of the kinematic viscosity to the diffusion coefficient is called the Schmidt number. For the DPD system with *s* = 0.5, the Schmidt number was estimated to be approximately 35 times larger than for the standard DPD (*s* = 2)^27^. Since simulations of homogalacturonan in a water solution required increased viscosity and reduced diffusion coefficient values, the values of *s* along with $\sigma$, $\gamma$ and conservative force coefficients were gradually modified in order to study the resulting changes in the desired target quantities.

### DPD simulations

In order to match the coarse-grained characteristics of MD simulations, the MD systems were reproduced using dissipative particle dynamics in a series of simulations. The DPD water model was simulated in a 10 × 10 × 10 DPD unit box (72 × 72 × 72 Å^3^), filled with water beads to obtain $\uprho =3.0$. The same water box was used to simulate single galacturonic acid molecules in a water solution. For non-bonded galacturonic acid interactions, the MD systems were reproduced with the same size of simulation boxes, as well as the number of galacturonic acid molecules and the corresponding CG water beads. Simplified models were initially created to match the parameters of the bonded interactions. Firstly, simple models of two galacturonic acid molecules connected with the distance bond were created to match the bond parameters (distribution of lengths between beads). Next, models of three galacturonic acid molecules connected with distance and angular bonds were created to parametrize the latter. Models were created for all combinations of dissociated and undissociated galacturonic acid molecules listed in section 2.3. After initial parametrization, the full-scale models of the molecular chains were reproduced to calibrate and refine the values of the bond parameters. All systems were initiated with 2000 warm-up, and were then equilibrated for at least 20,000 steps, with the trajectories and velocities saved after every 200 steps. Each system simulation was repeated at least 10 times. The equations of motion of the DPD beads were integrated using a modified velocity Verlet scheme 26. Random numbers for stochastic forces calculations were generated using a Mersenne Twister pseudo-random generator. The cell lists of bead pairs within a given cut-off distance of each other were refreshed every 8 time steps. The simulation framework was implemented as a hand-written and multi-thread code using the C++ programming language. The code architecture was optimized to perform computations using GPUs. This was achieved by including the OpenCL open-source library (Khronos Group, USA). The project was organized and compiled with the Microsoft Visual Studio Version 16.4.0 (Microsoft Corporation, USA). The access to the code repository will be shared upon the readers request.

## Results and discussion

### Water parametrization: calibration of the DPD model

The aim of this work was to establish the calibration scheme for the DPD system describing basic pectic carbohydrate—homogalacturonan. In order to achieve this goal, the dissipative particle dynamics systems were related to reference models created using atomistic molecular dynamics simulations. Three statistics were used to compare and match both techniques—radial distribution functions, particle velocities and diffusion coefficients. The calibration procedure involved changes in model parameters until the best match between the resulting statistics was obtained. The water model is important and can have a major impact on the resulting properties of the system^45^. Therefore, the initial step of model calibration required a match to be made between the basic water models for both techniques.

**Figure 3:**
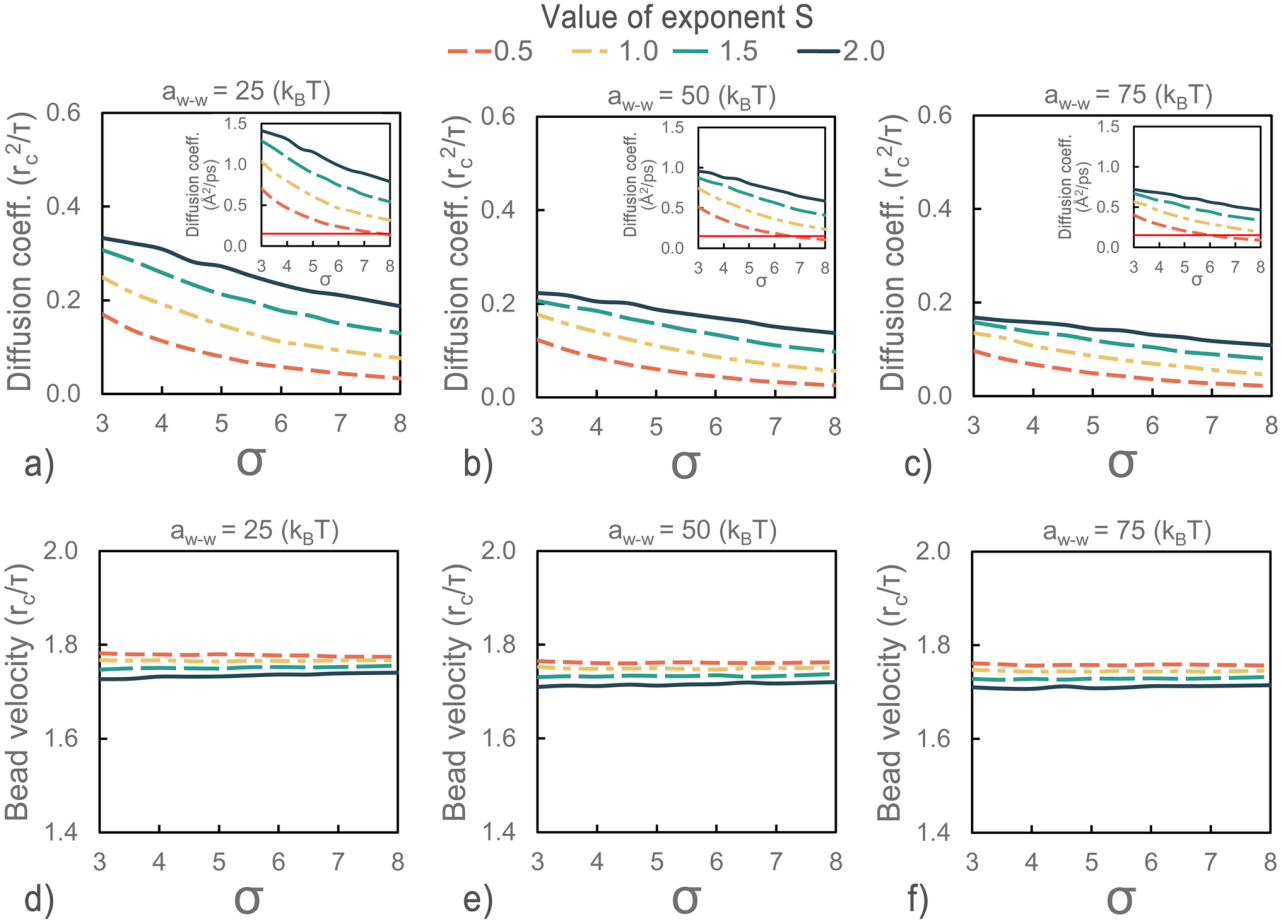
Parameter space plots showing changes in the diffusion coefficient and bead velocities with respect to σ and γ (obeying the following conditions: ${\upsigma }^{2}=2\upgamma {\mathrm{k}}_{\mathrm{B}}\mathrm{T}$) as well as values of the exponent s (from the weight function of dissipative and random forces), at three values of the conservative repulsion force coefficient. Sub-plots show the diffusion coefficients in real units after time scale calibration based on the average velocities of DPD and coarse-grained MD water beads. The red horizontal line at the sub-plots shows the value of the diffusion coefficient of the target MD system.

In the case of the DPD water model this could be accomplished through modifications of three parameters:$\upsigma$, $\upgamma$ and the water bead repulsive force coefficient ${\mathrm{a}}_{{\left({\mathrm{H}}_{2}\mathrm{O}\right)}_{4}}$. However, a comparison of the particle velocities and diffusion coefficient values required the establishment of the time scale of the DPD system. With the calibration of the time scale of the DPD system, the resulting parameters gain physical meaningfulness and could be compared with the MD model. For this purpose, a series of simulations were carried out which provided parameter space plots that showed the changes in the desired target quantities with respect to the values of the three modified parameters (Fig. 3).

The parametric analysis showed that changes in the values of the random and dissipative force coefficients ($\upsigma$ and $\upgamma$ ) had a large impact on the diffusion rate of the water beads, at the same time causing only fractional changes in their velocities. The diffusion rate of the water beads decreased with a corresponding increase in σ and γ. For the same combinations of values of s, $\upsigma$ and $\upgamma$ an increase in repulsion forces decreased the diffusion rate of the particles (clearly visible through lowering the lines for the same values of exponent s in Fig. 3a–c). This trend was also reported by Pivkin and Karniadakis^52^ who showed an increasing trend of total viscosity and a decreasing trend for the self-diffusion coefficient with the conservative force parameter. An increase in bead repulsion forces decreased the diffusivity of the system, however, and only caused a slight decrease in the velocities of the beads. Finally, the decrease in values of the exponent s resulted in a clear and distinct decrease of the diffusion rate of the water beads. Although the changes were fractional, the exponent s also showed the highest impact on the bead velocities among all of the parameters tested. The decrease in the s value caused a slight increase in the average velocities of the DPD water molecules.

Using the results of the parametric analysis, it was possible to provide the time scale of the DPD systems. The most common calibration approach in DPD relies on matching the diffusion constant of the DPD simulation with the experimental reference values^25^. In this technique, the diffusion constant obtained is compared to that of the molecule of interest in order to determine the simulation time scale. In the case of this study this would be expressed by the following relationship: ${D}_{DPD}\frac{{r}_{c}^{2}}{\tau }={D}_{MD}$ (where ${D}_{DPD}$ is the diffusion coefficient of the DPD system expressed in reduced units, ${D}_{MD}$ is the diffusion coefficient of the coarse-grained MD system expressed in real units, with ${r}_{c}$ and $\tau$ expressed in real units). When we match the diffusivities of the DPD beads obtained for a set of the commonly applied parameters ($s=2.0$, $\sigma =3.0$, $\gamma =4.5$, ${a}_{w-w}=25$, gives ${D}_{DPD}=0.332$, from Fig. 3a) and coarse-grained MD water molecules (${D}_{MD}=0.151\, {\AA }^{2}/\text{ps}$) the resulting time scale is equal to $\tau =113.97\,\text{ps}$. The calculation of the DPD bead velocities using $\tau =113.97\,\text{ps}$ provides values that differ from the values of the reference system by at least an order of magnitude (${\mathrm{v}}_{\mathrm{DPD}}=0.108\,\mathrm{\AA }/\mathrm{ps}$, with ${\mathrm{v}}_{\mathrm{MD}}=1.021\,\mathrm{\AA }/\mathrm{ps}$), indicating that the transport properties of the simulated system are lost. Moreover, considering the obtained results for parameter space analysis, which showed that *s* along with $\sigma$, $\gamma$ and the conservative force coefficient had an almost negligible impact on the velocities of the DPD beads, it is no longer possible to match the particle velocities without changes to the RDF or diffusion coefficient.

The calibration of the time scale is a common issue with DPD systems. In most cases models are calibrated for a specific application. This means that the parameters of the model are modified to obtain physically relevant values for the quantities of interest that describe the target system with a possible disparity between other physical quantities.

**Figure 4:**
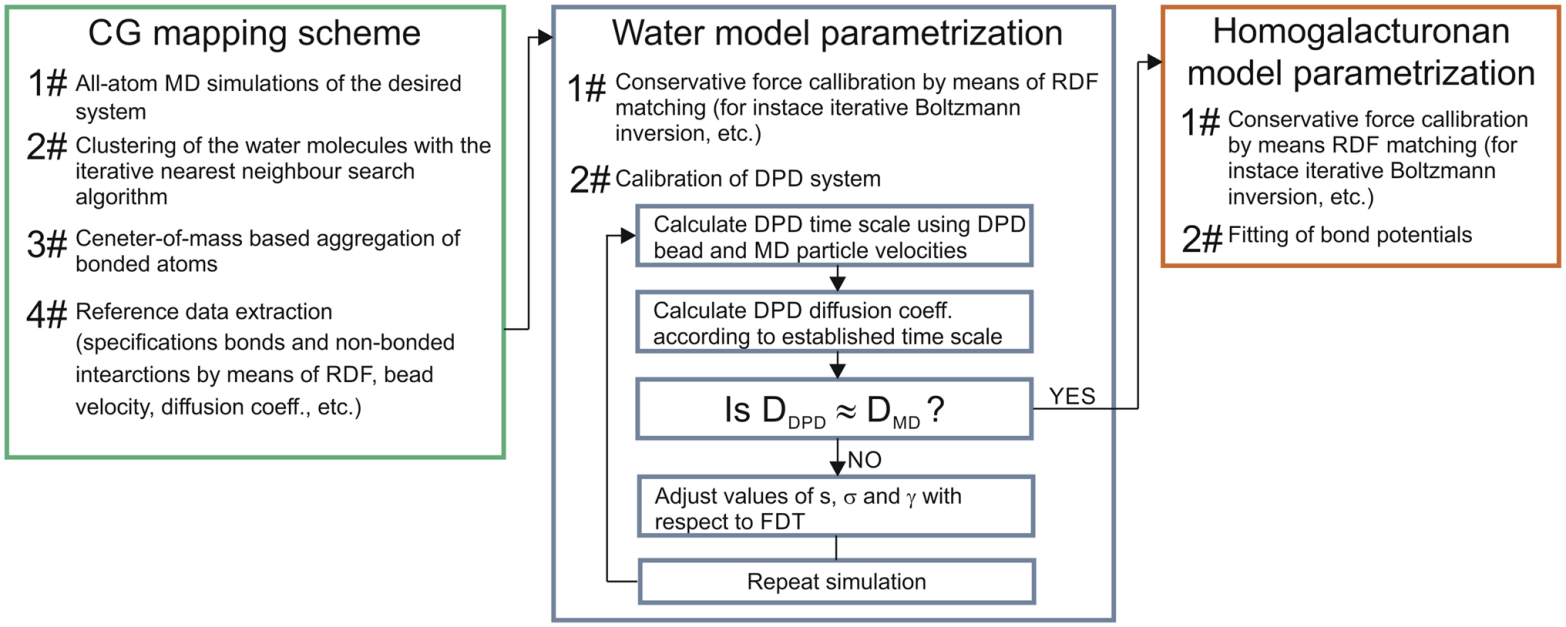
The schematic representation of the DPD force field parametrization work flow.

In this study, an alternative strategy of searching for an optimal parameter set for DPD simulations was proposed, which enables the preservation of the basic molecular statistics of the reference system. The schematic block representation of the parametrization scheme on Fig. 4.

**Figure 5:**
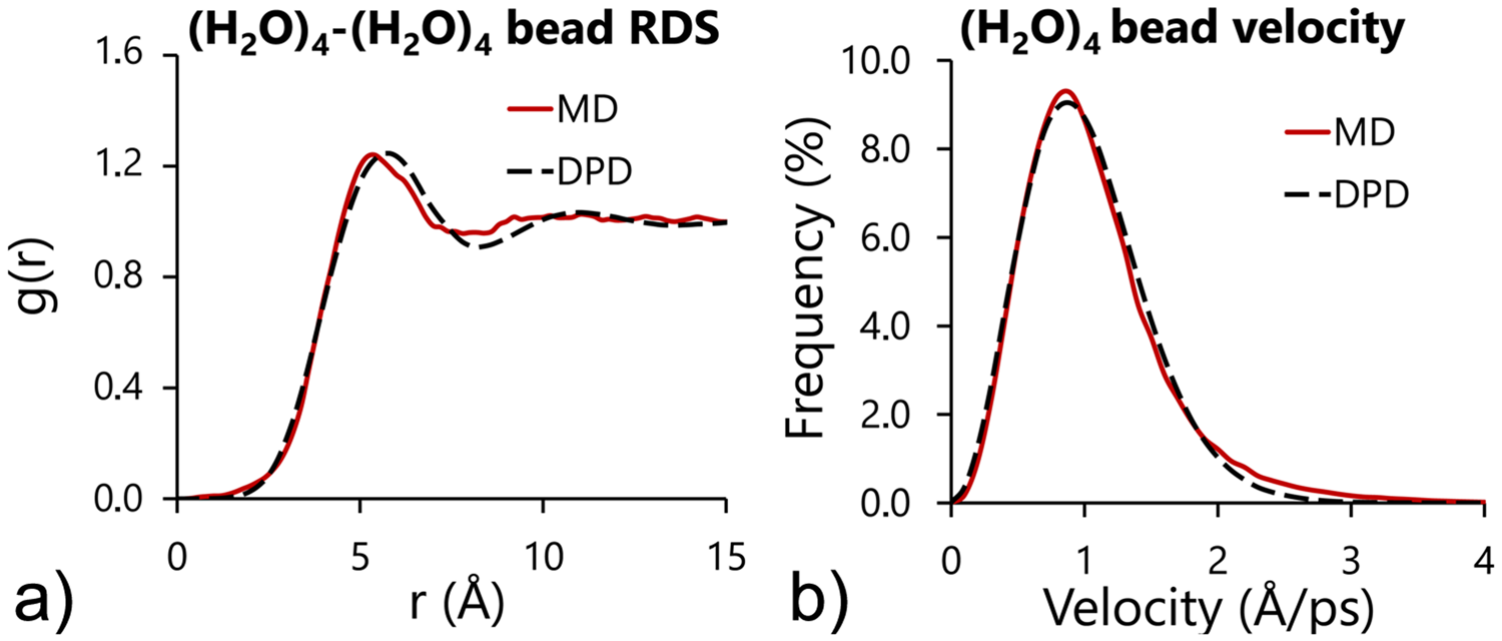
Results of the DPD model calibration compared with the MD reference model: (**a**) radial distribution function and (**b**) velocity distribution for water beads.

The parameter analysis showed that for ${\upsigma }^{2}=2\upgamma {\mathrm{k}}_{\mathrm{B}}\mathrm{T}$ the velocity of the water beads is almost independent of the s, $\upsigma$, $\upgamma$ and ${\mathrm{a}}_{{\left({\mathrm{H}}_{2}\mathrm{O}\right)}_{4}}$ values. Moreover, since the RDF is solely determined by the conservative force^53^, in the first step of system calibration, the repulsion force coefficient for water beads ${\mathrm{a}}_{{\left({\mathrm{H}}_{2}\mathrm{O}\right)}_{4}}$ was adjusted to match the MD and DPD radial distribution functions (Fig. 5a). The best match for the RDF shapes was obtained for ${\mathrm{a}}_{{\left({\mathrm{H}}_{2}\mathrm{O}\right)}_{4}}=50.0$. It should be emphasized that the aim of this study was to provide an alternative time scaling procedure and new algorithm for calculating the RDF, velocities and diffusion coefficients from the MD atomistic trajectories mapped to the coarse-grained level, which would serve as the target properties for the optimization of the DPD system. The procedures of fitting the mean force potentials such as the iterative Boltzmann inversion are not within the scope of this work, however, it is important to point out that the mapping procedure provided is fully compatible with such algorithms.

In the next step of model calibration, the time scale of the DPD system was established by matching the average velocity of DPD and the atomistic MD water particles using the following expression $\uptau =\frac{{\mathrm{v}}_{\mathrm{DPD}}}{{\mathrm{v}}_{\mathrm{MD}}}{\mathrm{r}}_{\mathrm{C}}$ (where ${\mathrm{v}}_{\mathrm{DPD}}$ is the average velocity of the DPD beads expressed in reduced units, and ${\mathrm{v}}_{\mathrm{MD}}$ is the average velocity of the MD particles expressed in real units, with $\tau$ and ${r}_{c}$ expressed in real units). With the time scale established, the DPD diffusion coefficient was rescaled to real units and compared with the results from MD simulations. The results of the procedure above applied to data from the parameter space analysis are shown on subplots with the time scale calibrated diffusion coefficients of the DPD beads expressed in real units (Fig. 3a–c). The red horizontal line from the sub-plots shows the value of the diffusion coefficient of the target MD system. The intersection of both lines indicates the optimal set of simulation parameters that provide the best match between MD and DPD diffusion coefficients. For ${\mathrm{a}}_{{\left({\mathrm{H}}_{2}\mathrm{O}\right)}_{4}}=50.0$ the best match between the MD and DPD diffusion coefficients was obtained for $\upsigma =6.86$ and $\upgamma =23.53$, when s = 0.5.

The calibration results based on ten simulation runs are shown in Table 1 and Fig. 5. The proposed parametrization approach allowed for the preservation of three characteristics of the atomistic MD system—the shape of the RDF of the water beads, the distribution of the velocities of the water beads and the diffusion coefficient of the system (Fig. 5a, b). The obtained time scale of the DPD system $\uptau =12.47$ ps and the corresponding time step were significantly lower than the time scales reported in other DPD studies, where scaling was based on the diffusion coefficient only^24,55^. The corresponding time step (12.47 fs) was also in good agreement with other coarse-grained simulation techniques and force fields (for instance with the MARTINI force field^56^), which again is in line with the findings of other researchers, which pointed out that the proper application of DPD requires a significant reduction in the time-step length with respect to commonly accepted values^49^.

**Table 1:** Calibration of the time scale of the DPD system based on ten data samples from the MD model.

Simulation no	Atomistic particle velocity (Å/ps)	DPD time scale τ (ps)	Atomistic diffusion coeff. (Å^2^/ps)	DPD diffusion coeff (Å^2^/ps)
1	1.018	12.501	0.144	0.151
2	1.023	12.455	0.137	0.149
3	1.024	12.443	0.139	0.149
4	1.019	12.517	0.155	0.160
5	1.022	12.474	0.155	0.152
6	1.019	12.506	0.155	0.146
7	1.023	12.478	0.154	0.151
8	1.020	12.472	0.145	0.150
9	1.019	12.461	0.158	0.149
10	1.022	12.394	0.165	0.150
Average	1.021	12.470	0.151	0.151

Particle velocities and diffusion coefficients are calculated for molecular clouds of MD water molecules. τ is a DPD time scale derived from the MD model based on particle velocity comparison. Results obtained for ${\mathrm{a}}_{{\left({\mathrm{H}}_{2}\mathrm{O}\right)}_{4}}=50.0$,$\upsigma =6.86$, $\upgamma =23.53$, s = 0.5.

As Boromand et al.^54^ noted previously, the random and dissipative forces have a crucial role in defining the dynamics of DPD and they can assume any value as long as they satisfy the dissipation-fluctuation relationship. However, the majority of reported studies use the commonly accepted values of 3.0 and 4.5 for random and dissipative parameters, respectively. The results of the current study showed that if the goal of the DPD system is to preserve both the structural and transport properties of the target system, then the values of exponent s, $\sigma$ and $\gamma$ cannot be taken freely. In fact, for $\upsigma =3.0$ and $\upgamma =4.5$ it was the case that all of the tested values of s and repulsive force coefficients simulations failed to match the target values of the transport properties. Also, the simulated systems did not match the diffusivity of the target system for the s values above 1.0, regardless of the value of the repulsive force coefficient, $\sigma$ or $\gamma$.

Moreover, among all of the values tested only at s = 0.5 was it possible to adjust the magnitudes of the random and dissipative forces to match the desired diffusivity, it should be noted that the exponent s can take any value from the set of positive real numbers. Therefore, the set of parameters provided is not unique. Other values of s (between 0.5 and 1.0 in the case of this DPD system for instance) are valid so long as it is possible to adjust the values of $\sigma$ or $\gamma$ to meet the desired matching criteria and provide physically meaningful results.

### Parametrization of non-bonded interactions between galacturonic acid molecules

**Figure 6:**
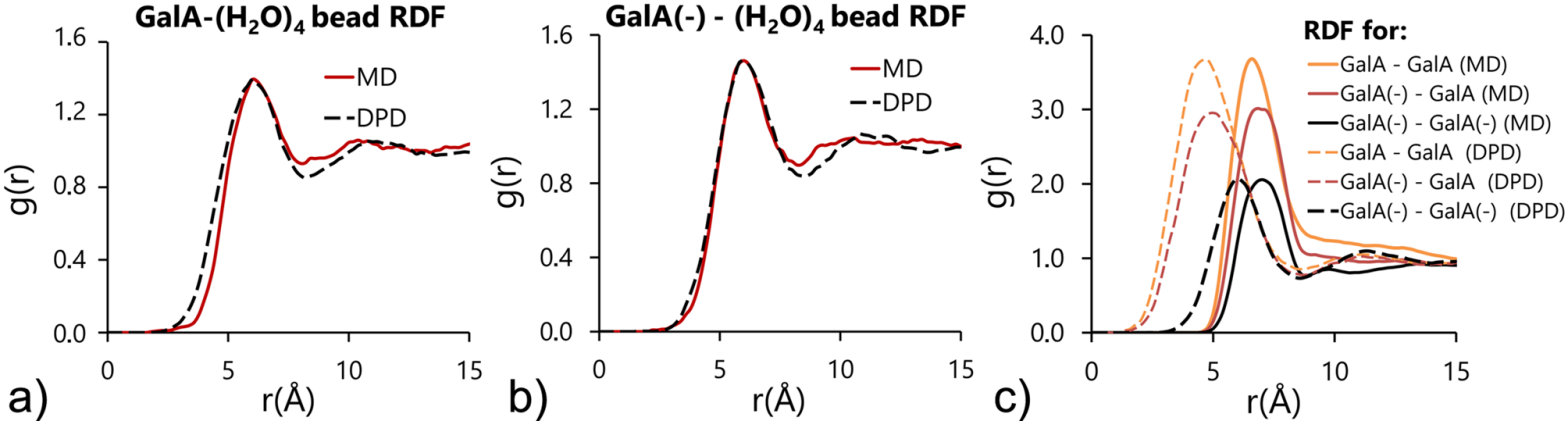
Radial distribution functions for non-bonded interactions of galacturonic acid molecules.

With a properly scaled water model, the non-bonded interactions of galacturonic acid molecules were parametrized based on a set of MD and DPD simulations described in sections 2.3 and 2.6. The parametrization scheme relied on RDF matching of galacturonic acid molecules interacting with the atomistic water molecules. Since the same values of $\sigma$ and $\upgamma$ were set for the entire DPD, the parametrization procedure was reduced to changes in the conservative repulsion force coefficient, until the best match with the target RDF was obtained. The results of parametrization are shown in Fig. 6. The values of the conservative force coefficients are gathered in Table 2.

**Table 2:** Values of the DPD conservative force interaction parameters *a*_*ij*_ (*k*_*B*_*T*) derived from MD for different interaction pairs.

	(H_2_O)_4_	GalA(−)	GalA
(H_2_O)_4_	50.0	–	–
GalA(-)	75.0	100.0	–
GalA	67.0	65.0	58.5

In general, the magnitude of the repulsion forces between galacturonic acid molecules and their environment was dependent on the charge of the molecule. For negatively charged molecules (GalA(−)) the self-repulsion and water repulsion forces were higher than they were for the molecules with a neutral charge (Table 2). The heights of the RDF peaks suggested the possibility of temporal associations of galacturonic acid molecules. This possibility was increased if the carboxyl group of at least one of the interacting molecules was protonated (undissociated). For GalA(−) molecules with deprotonated carboxyl groups this likelihood was lower but despite the highest self-repulsion coefficient, short-term associations of GalA(-) were still possible. This corresponded with the observed properties of pectic polysaccharides, which showed greater gelling abilities under low pH conditions^18^.

For galacturonic acid self-interactions the quality of the match between the RDF shapes differed depending on the type of simulated molecule. The relatively favourable correspondence of the RDF with the MD results was obtained with respect to the height and shape of the curves. However, all of the DPD curves were shifted towards the centres of the interacting beads. This was a consequence of the implementation of the DPD force field soft potentials, which in some circumstances allow particles to pass through each other. This is impossible in an MD simulation where hard Lennard–Jones potentials are applied at the cost of smaller time steps. Nevertheless, the resulting radial distribution functions were considered to be good representations of the target system.

### Parametrizations of bonded interactions

**Figure 7:**
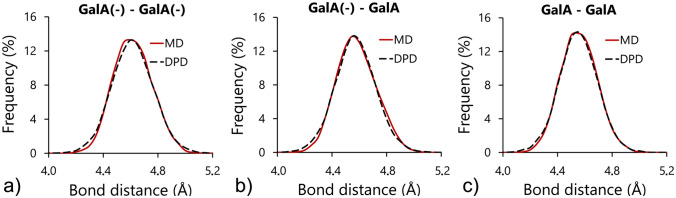
The dissipative particle dynamics best fit distributions of lengths for distance bonds compared with the corresponding data obtained using molecular dynamics simulations.

**Figure 8:**
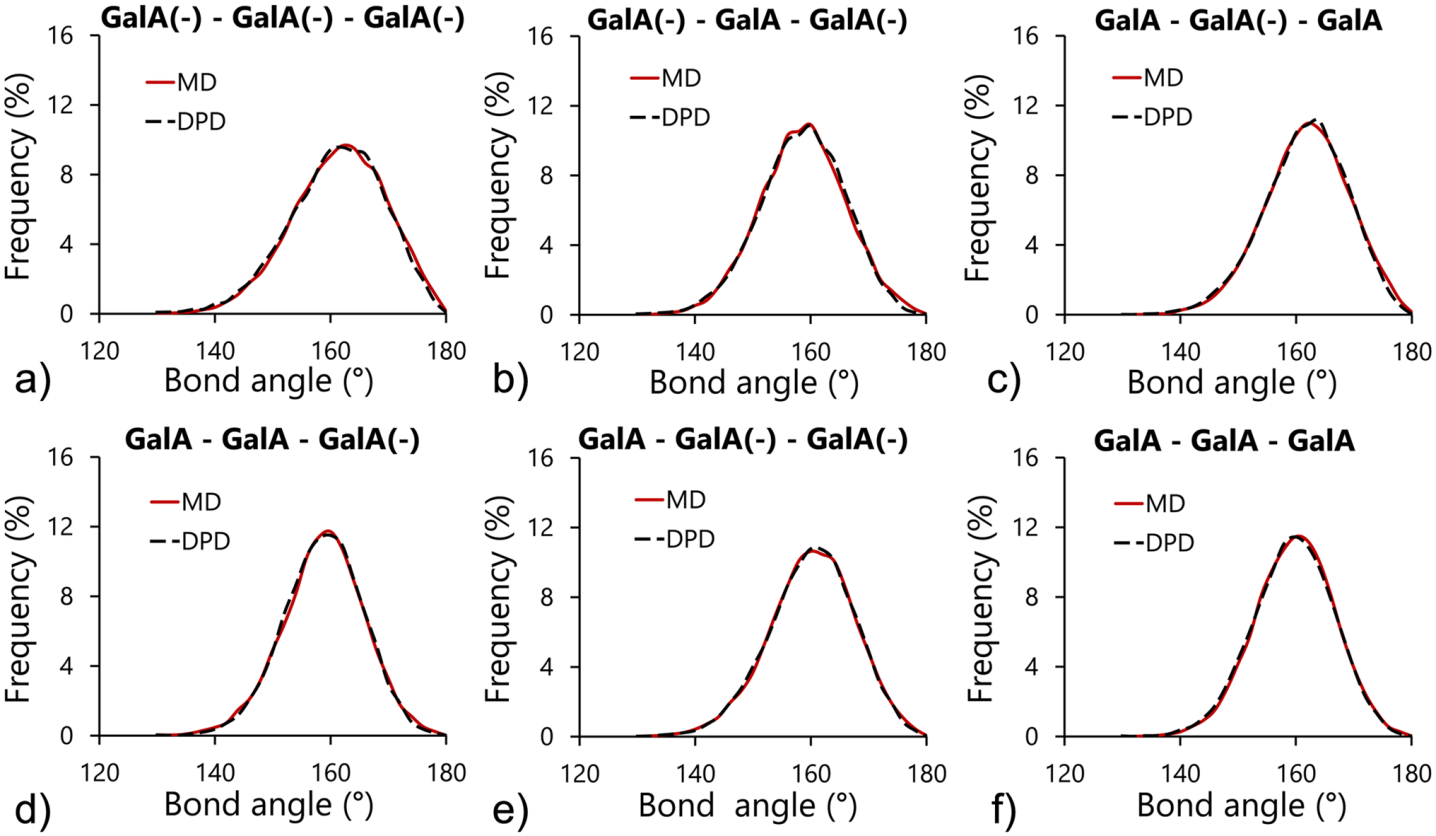
The dissipative particle dynamics best fit distributions of bond angles compared with the corresponding data obtained using molecular dynamics simulations.

The final step in homogalacturonan chain modelling involved the parametrization of bonded interactions between galacturonic acid molecules. The DPD bonded parameters were tuned until the values of the structural parameters of coarse-grained molecules (distances and angles between molecules) were close to the data obtained using MD simulations. Tables 3 and 4 summarize the results of fitting for distance and angular bonds. The obtained quality of fit of distance and angle distributions for all combinations of bonds are shown in Figs. 7 and 8.

**Table 3:** The DPD distance bond parameters derived from molecular dynamics simulations.

Bond length	${k}_{s}$ (*k*_*B*_*T*/*r*_*c*_^2^)	${l}_{0}$ (r_c_)
GalA(−)–GalA(−)	2,700.0	0.63 (4.57 nm)
GalA(−)–GalA	2,920.0	0.63 (4.56 nm)
GalA–GalA	3,375.0	0.62 (4.55 nm)

**Table 4:** The DPD angular bond parameters derived from molecular dynamics simulations.

Angular bond	${k}_{A}$ (*k*_*B*_*T*/rad^2^)	${\theta }_{0}$ (rad)
GalA(−)–GalA(−)–GalA(−)	17.0	2.81 (161.52°)
GalA–GalA(−)–GalA(−)	25.0	2.79 (160.15°)
GalA(−)–GalA–GalA(−)	26.0	2.76 (158.67°)
GalA–GalA–GalA(−)	32.0	2.77 (158.78°)
GalA–GalA(−)–GalA	24.0	2.82 (161.86°)
GalA–GalA–GalA	29.0	2.83 (162.66°)

Panczyk et al.^22^ studied the effect of the orientation of glycosidic linkages between residues on the conformation of longer, oligo- or polymeric saccharide chains. The obtained results indicated that the chemical state of the carboxyl group was not relevant in the context of the conformation of longer saccharide chains. Both the location of the main minima of free energy and the dimension of the available conformational phase space remained unchanged, and independent of whether the carboxyl group was protonated, deprotonated or esterified. This stability of the 1–4 glycosidic linkages between galacturonic acid molecules was reproduced in a coarse-grained model of homogalacturonan presented in the current study. This was confirmed by the relatively low variations in the values of the rest lengths and angles (${\mathrm{l}}_{0}$ and ${\uptheta }_{0}$) for different combinations of length and angular bonds (Tables 3 and 4). The stiffness of the bonds was expressed by *k*_*S*_ and *k*_*A*_ which varied with respect to the repulsion force of the linked beads. The higher the repulsion force, the lower the stiffness of the bonds.

The resulting bond parameters provided very well matched distributions of the structural parameters of simulated bonds (Figs. 7 and 8). A similar fitting procedure was applied by Sepehr and Paddison^4^ for the simulations of hydrated perfluorosulphonic acid ionomers in water solutions. The authors reported that at given time steps, i.e. the relatively long time steps used (more than ten times longer than those used in this study) the stiffness parameters of the bonds allowed for a match with the MD characteristics only qualitatively. The exact replication of the MD structures required the stiffness of the bonds to become too large for applied time stepping, resulting in unstable simulations. In this study, the high quality of fit was attributed to the application of relatively short simulation time steps. It was also concluded that short-time stepping is necessary in order to preserve the original conformations of polymeric chains in terms of the distance and angular bond characteristics.

### Simulations of homogalacturonan chains

Simulations were performed on the system of homogalacturonan chains in water solutions. The simulation consisted of 100,000 beads with 7,560 being galacturonic acid molecules, all with a protonated carboxyl group (GalA). This corresponded to a 0.3 mol concentration of galacturonic acid in water. Homogalacturonan chains with uniform lengths equal to 35 units of GalA, were initially randomly generated in a 231 × 231 × 231 Å simulation box. The simulated box contained 216 homogalacturonan chains in total.

**Figure 9:**
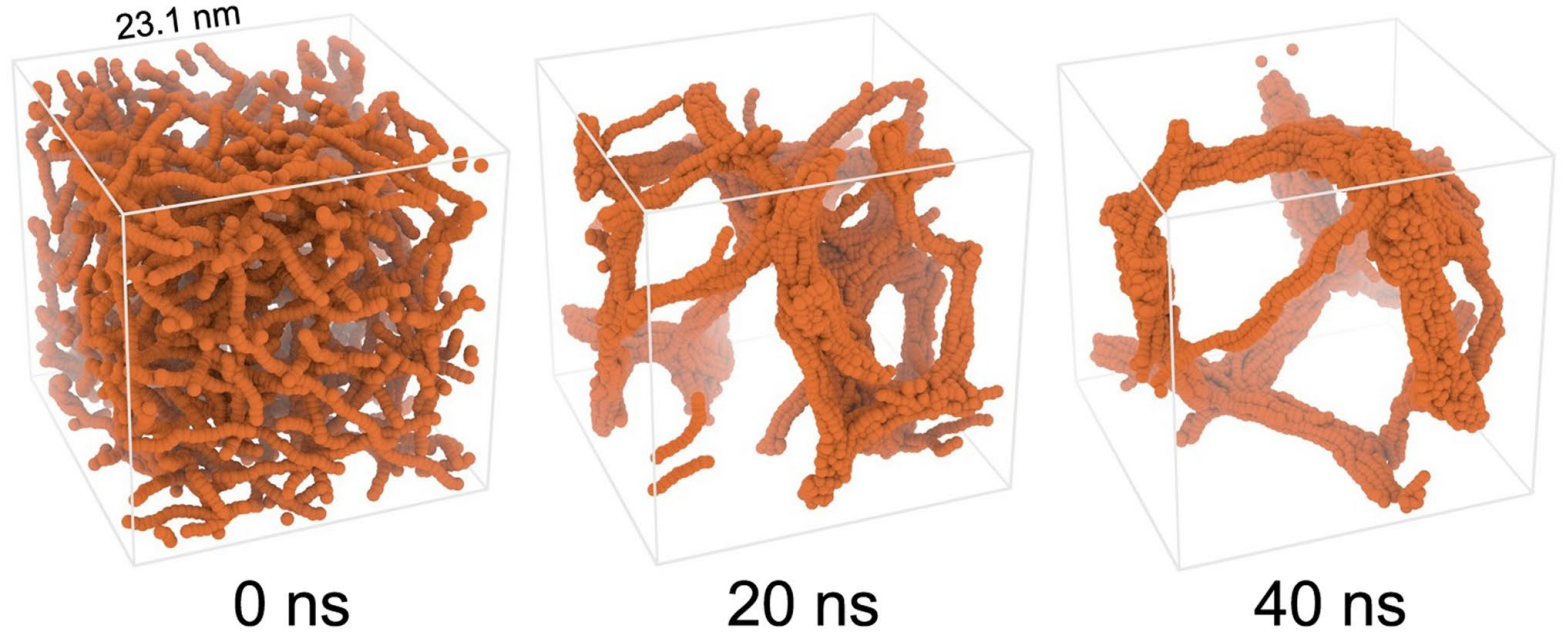
Results of DPD simulations based on the proposed parametrization approach showing three stages of molecular self-assembly of homogalacturonan chains (visualization using Blender 2.79, Blender Foundation, Amsterdam, Netherlands, https://www.blender.org/).

The system was equilibrated for 1 680 000 steps which was equal to 40 ns of simulation time. Figure 9 shows three snapshots from the conducted simulation. The resulting DPD trajectories showed that randomly dispersed homogalacturonan chains showed a tendency to aggregate into highly organized 3D structures. The final structure resembled a three-dimensional network created by tightly associated homogalacturonan chains organized into thick fibres. The ability of pectic polysaccharides to form structural networks was reported by other researchers, who indicated the hydrogen bonding of carboxyl groups as a possible self-aggregation mechanism^18,56^. Previous studies also showed that the homogalacturonan-rich fraction of pectin extracted from fresh fruits and vegetables such as carrot, apple or pear, form a regular interlinked network when they dry out on mica^23,58,59^. It should be noted that atomic force microscopy studies were limited to 2D observations and that the simulated system was still smaller than the structures observed using AFM. Other studies demonstrated that the homogalacturonan rich fraction of pectin has unique thixotropic rheological properties^18,60,61^. Thixotropy arises from the structural decomposition and regeneration of polymeric particles as a function of time. The formation of three-dimensional networks similar to those reported in this study may play an essential role in the thixotropic properties shown by pectic polysaccharides rich in homogalacturonan chains.

## Conclusions

In this study a new method for the parametrization of the DPD force field was demonstrated with the example of basic pectic polysaccharide–homogalacturonan. Parametrization relied on the extraction of atomistic parameters from molecular dynamics using a proposed molecule aggregation algorithm based on an iterative nearest neighbour search. Moreover, a new approach to a time-scale calibration scheme based on matching the average velocities of particles was presented. The study demonstrated the successful application of the proposed parametrization method which enabled the reproduction of the shapes of radial distribution functions, particle velocities and diffusivity of an atomistic molecular dynamics model using a dissipative particle dynamics force field. The DPD force field was able to reproduce the essential structural features of homogalacturonan molecular chains by means of distance and angular bond characteristics, which closely matched the MD results. Firstly, the fully parametrized coarse-grained DPD model of homogalacturonan chains in a water solution was presented. The force field parameters indicated the possibility of the self-aggregation of galacturonic acid molecules via the hydrogen bonding of carboxyl groups. Further simulations confirmed these suppositions providing the first insights into the three-dimensional structures created by large assemblies of homogalacturonan in water solutions. The initial simulations will be followed by a more extensive exploration of the obtained force field. The presented parameterized model of homogalacturonan will become a promising tool in simulating the effects of chain lengths, galacturonic acid concentrations and different protonation patterns of carboxyl groups with a self-assembly mechanism.

## References

[1] Lee M-T, Mao R, Vishnyakov A, Neimark AV (2016). Parametrization of chain molecules in dissipative particle dynamics. J. Phys. Chem..

[2] Hoogerbrugge PJ, Koelman JMVA (1992). Simulating microscopic hydrodynamic phenomena with dissipative particle dynamics. Europhys. Lett..

[3] Español P, Warren P (1995). Statistical mechanics of dissipative particle dynamics. Europhys. Lett..

[4] Sepehr F, Paddison SJ (2016). Dissipative particle dynamics interaction parameters from *ad initio* calculations. Chem. Phys. Lett..

[5] Iacovella CR, Keys AS, Glotzer SC (2011). Self-assembly of soft-matter quasicrystals and their approximants. Proc. Natl. Acad. Sci. USA.

[6] Nguyen HD, Hall CK (2004). Molecular dynamics simulations of spontaneous fibril formation by random-coil peptides. Proc. Natl. Acad. Sci. USA.

[7] Soto-Figueroa C, Rodríguez-Hidalgo M, Vicente L (2012). Dissipative particle dynamics simulation of the micellization–demicellization process and micellar shuttle of a diblock copolymer in a biphasic system (water/ionic-liquid). Soft Matter.

[8] Wang Y, Li B, Zhou Y, Lu Z, Yan D (2013). Dissipative particle dynamics simulation study on the mechanisms of self-assembly of large multimolecular micelles from amphiphilic dendritic multiarm copolymers. Soft Matter.

[9] Moshfegh A, Jabbarzadeh A (2015). Dissipative particle dynamics: Effects of parameterization and thermostating schemes on rheology. Soft Mater..

[10] Groot RD, Warren PB (1997). Dissipative particle dynamics: Bridging the gap between atomistic and mesoscopic simulation. J. Chem. Phys..

[11] Tschöp W, Kremer K, Batoulis J, Bürger T, Hahn O (1998). Simulation of polymer melts. I. Coarse-graining procedure for polycarbonates. Acta Polym..

[12] Vishnyakov A, Neimark AV (2014). Self-assembly in Nafion membranes upon hydration: Water mobility and adsorption isotherms. J. Phys. Chem. B.

[13] Keaveny EE, Pivkin IV, Maxey M, Karniadakis GE (2005). A comparative study between dissipative particle dynamics and molecular dynamics for simple- and complex-geometry flows. J. Chem. Phys..

[14] Li Z, Bian X, Caswell B, Karniadakis G (2014). Construction of dissipative particle dynamics models for complex fluids via the Mori–Zwanzig formulation. Soft Matter.

[15] Morris ER, Rees DA, Thom D, Boyd J (1978). Chiroptical and stoichiometric evidence of a specific, primary dimerisation process in alginate gelation. Carbohydr. Res..

[16] Braccini I, Pérez S (2001). Molecular basis of Ca^2+^-induced gelation in alginates and pectins: The egg-box model revisited. Biomacromol.

[17] Walkinshaw MD, Arnot S (1981). Conformations and Interactions of pectins: I. X-ray diffraction analyses of sodium pectate in neutral and acidified forms. J. Mol. Biol..

[18] Gawkowska D, Cieśla J, Zdunek A, Cybulska J (2019). Cross-linking of diluted alkali-soluble pectin from apple (*Malus domestica* fruit) in different acid-base conditions. Food Hydrocoll..

[19] Manunza B, Deiana S, Pintore M, Gessa C (1997). A molecular dynamics investigation on the occurrence of helices in polygalacturonic acid. J. Mol. Struct..

[20] Manunza B, Deiana S, Pintore M, Gessa C (1997). Molecular dynamics study of polygalacturonic acid chains in aqueous solution. Carbohydr. Res..

[21] Makshakova ON, Gorshkova TA, Mikshina PV, Zuev YF, Perez S (2017). Metrics of rhamnogalacturonan I with β-(1→4)-linked galactan side chains and structural basis for its self-aggregation. Carbohydr. Polym..

[22] Panczyk K, Gaweda K, Drach M, Plazinski W (2018). Extension of the GROMOS 56a6CARBO/CARBO-R force field for charged, protonated, and esterified uronates. J. Phys. Chem. B.

[23] Pieczywek PM, Kozioł A, Płaziński W, Cybulska J, Zdunek A (2020). Resolving the nanostructure of sodium carbonate extracted pectins (DASP) from apple cell walls with atomic force microscopy and molecular dynamics. Food Hydrocoll..

[24] Braccini I, Grasso RP, Pérez S (1999). Conformational and configurational features of acidic polysaccharides and their interactions with calcium ions: A molecular modeling investigation. Carbohydr. Res..

[25] Groot RD, Rabone KL (2011). Mesoscopic simulation of cell membrane damage, morphology change and rupture by nonionic surfactants. Biophys. J.

[26] Moeendarbary E, Ng TY, Zangeneh M (2009). Dissipative particle dynamics: Introduction, methodology and complex fluid applications—A review. Int. J. Appl. Mech..

[27] Fan X, Phan-Thien N, Chen S, Wu X, Ng TY (2006). Simulating flow of DNA suspension using dissipative particle dynamics. Phys. Fluids.

[28] Symeonidis V, Karniadakis GE, Caswell B (2006). Schmidt number effects in dissipative particle dynamics simulation of polymers. J. Chem. Phys..

[29] Abraham MJ, Murtola T, Schulz R, Pàll S, Smith JC, Hess B, Lindahl E (2015). GROMACS: High performance molecular simulations through multi-level parallelism from laptops to supercomputers. SoftwareX.

[30] Hansen HS, Hünenberger PH (2011). A reoptimized GROMOS force field for hexopyranose-based carbohydrates accounting for the relative free energies of ring conformers, anomers, epimers, hydroxymethyl rotamers, and glycosidic linkage conformers. J. Comput. Chem..

[31] Płaziński W, Lonardi A, Hünenberger PH (2016). Revision of the GROMOS 56A6_CARBO_ force field: Improving the description of ring-conformational equilibria in hexopyranose-based carbohydrates chains. J. Comput. Chem..

[32] Berendsen HJC, Postma JPM, van Gunsteren WF, Hermans J, Pullman B (1981). Interaction models for water in relation to protein hydration. Intermolecular Forces.

[33] Bussi G, Donadio D, Parrinello M (2007). Canonical sampling through velocity rescaling. J. Chem. Phys..

[34] Parrinello M, Rahman A (1981). Polymorphic transitions in single crystals: A new molecular dynamics method. J. Appl. Phys..

[35] Hockney RW (1970). Potential calculation and some applications. Methods Comput. Phys..

[36] Hess B (2008). P-LINCS: A parallel linear constraint solver for molecular simulation. J. Chem. Theory Comput..

[37] Hess B, Bekker H, Berendsen HJC, Fraaije JGEM (1997). LINCS: A linear constraint solver for molecular simulations. J. Comput. Chem..

[38] Berendsen HJC, van Gunsteren WF, Zwinderman HRJ, Geurtsen RG (1986). Simulations of proteins in water. Ann. N. Y. Acad. Sci..

[39] Barker JA, Watts RO (1973). Monte Carlo studies of the dielectric properties of water-like models. Mol. Phys..

[40] Tironi IG, Sperb R, Smith PE, van Gunsteren WF (1995). A generalized reaction field method for molecular dynamics simulations. J. Chem. Phys..

[41] Heinz TN, van Gunsteren WF, Hünenberger PH (2001). Comparison of four methods to compute the dielectric permittivity of liquids from molecular dynamics simulations. J. Chem. Phys..

[42] Chiu S, Scott HL, Jakobsson EA (2010). Coarse-grained model based on morse potential for water and n-alkanes. J. Chem. Theory Comput..

[43] Van Hoof B, Markvoort AJ, Van Santen R, Hilbers PJ (2011). The CUMULUS coarse graining method: Transferable potentials for water and solutes. J. Phys. Chem. B.

[44] Hadley KR, McCabe C (2012). Coarse-grained molecular models of water: A review. Mol. Simul..

[45] Hadley KR, McCabe C (2010). On the investigation of coarse-grained models for water: Balancing computational efficiency and the retention of structural properties. J. Phys. Chem. B.

[46] Vaz RV, Gomes JRB, Silva CM (2016). Molecular dynamics simulation of diffusion coefficients and structural properties of ketones in supercritical CO_2_ at infinite dilution. J. Supercrit. Fluids.

[47] Pedretti A, Mazzolari A, Vistoli G, Testa B (2018). MetaQSAR: An integrated database engine to manage and analyze metabolic data. J. Med. Chem..

[48] Leimkuhler B, Shang X (2015). On the numerical treatment of dissipative particle dynamics and related systems. J. Comput. Phys..

[49] Hafskjold B, Liew CC, Shinoda W (2004). Can such long time steps really be used in dissipative particle dynamics simulations?. Mol Simulat..

[50] Krafnick RC, Garcia A (2015). Efficient Schmidt number scaling in dissipative particle dynamics. J. Chem. Phys..

[51] Azhar M, Greiner A, Korvink JG, Kauzlarić D (2016). Dissipative particle dynamics of diffusion-NMR requires high Schmidt-numbers. J. Chem. Phys..

[52] Pivkin IV, Karniadakis GE (2006). Coarse-graining limits in open and wall-bounded dissipative particle dynamics systems. J. Chem. Phys..

[53] Zohravi E, Shirani E, Pishevar A (2018). Influence of the conservative force on transport coefficients in the DPD method. Mol. Simul..

[54] Boromand A, Jamali S, Maia JM (2015). Viscosity measurements techniques in dissipative particle dynamics. Comput. Phys. Commun..

[55] Vaiwala R, Jadhav S, Taokar R (2017). Four-to-one coarse-grained polarizable water model for dissipative particle dynamics. Mol. Simul..

[56] Winger M, Trzesniak D, Baron R, van Gunsteren WF (2009). On using a too large integration time step in molecular dynamics simulations of coarse-grained molecular models. Phys. Chem. Chem. Phys..

[57] Huynh UTD, Lerbret A, Neiers F, Chambin O, Assifaoui A (2016). Binding of divalent cations to polygalacturonate: A mechanism driven by the hydration water. J. Phys. Chem. B.

[58] Zdunek A, Kozioł A, Pieczywek PM, Cybulska J (2014). Evaluation of the nanostructure of pectin, hemicellulose and cellulose in the cell walls of pears of different texture and firmness. Food Bioprocess Technol..

[59] Cybulska J, Zdunek A, Kozioł A (2015). The self-assembled network and physiological degradation of pectins in carrot cell walls. Food Hydrocoll..

[60] Mierczyńska J, Cybulska J, Pieczywek PM, Zdunek A (2015). Effect of storage on rheology of water-soluble, chelate-soluble and diluted alkali-soluble pectin in carrot cell walls. Food Bioprocess Technol..

[61] Gawkowska D, Cybulska J, Zdunek A (2018). Cross-linking of sodium carbonate-soluble pectins from apple by zinc ions. Carbohydr. Polym..

